# Probing Ionic Liquid Aqueous Solutions Using Temperature of Maximum Density Isotope Effects

**DOI:** 10.3390/molecules18043703

**Published:** 2013-03-25

**Authors:** Mohammad Tariq, José M. S. S. Esperança, Luís P. N. Rebelo, José N. Canongia Lopes

**Affiliations:** 1 Instituto de Tecnologia Química e Biológica (www.itqb.unl.pt), Universidade Nova de Lisboa, Av. da República, Oeiras 2780-157, Portugal; 2 Centro de Química Estrutural, Instituto Superior Tecnico, Lisboa 1049-001, Portugal

**Keywords:** water TMD, ionic liquids, isotope effects

## Abstract

This work is a new development of an extensive research program that is investigating for the first time shifts in the temperature of maximum density (TMD) of aqueous solutions caused by ionic liquid solutes. In the present case we have compared the shifts caused by three ionic liquid solutes with a common cation—1-ethyl-3-methylimidazolium coupled with acetate, ethylsulfate and tetracyanoborate anions—in normal and deuterated water solutions. The observed differences are discussed in terms of the nature of the corresponding anion-water interactions.

## 1. Introduction

Water is a complex substance whose unique properties derive from a balance between its ability to perform multiple hydrogen bonds (HBs) and its rather small molar volume and symmetric nature. One of the most well known consequences of such subtle balance is the open structure of ice and the associated density increase upon melting (+9%). Even in the liquid state such open hydrogen-bonded structure is only progressively lost: pure water continues to contract until a temperature of maximum density (TMD) is reached around 4 °C (the density increases +0.013% between 0 and 4 °C) [1].

A similar state of affairs also applies to deuterated water. However, given the different nature of the deuterium bonds (DBs), the melting point temperature and the TMD of D_2_O only occur at 3.82 and 11.21 °C, respectively [2,3].

One way to probe the nature of the hydrogen-bonded structure of aqueous solutions is to measure the shifts in the TMD, Δθ = TMD (aqueous solution)-TMD (pure water), of (normal or heavy) water solutions: solutes that promote more stable HB/DB networks should yield solutions with higher TMDs, whereas those that break the HB/DB network should decrease the TMD value.

Recently we have extensively measured the TMD shifts in normal water solutions caused by different ionic liquid (IL) solutes [4]. The diversity of this novel class of compounds allowed us to investigate in a systematic manner the different types of effect—hydrophobic, electrostatic, hydrogen-bonding—that can contribute to the overall TMD shifts.

All studied IL aqueous solutions exhibited negative shifts obeying the Despretz rule [5,6] that states that the TMD shifts should be proportional to the amount and nature of the added solute. Given the relatively large molar volume and positive thermal expansion coefficients of ionic liquids (the cations are generally bulky organic ions) such negative deviations are only to be expected: any stabilization of the original HB network of water caused by any specific ion-water interaction will be countered and superseded by the insertion of bulky ions in the midst of the water molecules. Even if the water molecules reorganize themselves around the large solute ions in a new structured network, its original ice-like open structure will be lost. Nevertheless the results have shown that different ionic liquids under analogous concentration conditions produce negative TMD shifts with very different slopes. Such differences are mainly anion-dependent and follow the hydrophilic/hydrophobic sequences generally used for ordering ionic liquid anions.

In order to further investigate the cause of such differences we have selected three ionic liquids with distinct hydrophilic/hydrophobic character and measured the TMD shifts of the corresponding solutions prepared with normal and deuterated water.

## 2. Experimental

### 2.1. Materials

The 3 ionic liquids used in the present work were purchased from different suppliers with the purity listed in Table 1. The table also shows the acronyms used throughout the work and the corresponding structural formula of the common cation. Prior to their use all ILs were dried at moderate temperatures (50–70 °C) under vacuum for 48 hours and their purity re-checked by ^1^H NMR. Millipore water was used for preparation of all aqueous (H_2_O) solutions. Deuterated water (Deuterium Oxide, D content of 99.9%) was purchased from Cambridge Isotope Laboratories, Inc. It was always handled under inert and dry atmosphere. All solutions were prepared gravimetrically using a Ohaus balance with ±0.00001 g precision. The uncertainty in the reported concentration values is ±0.0001 molal.

### 2.2. Methods

The densities of the ionic liquid aqueous solutions and of pure water (both normal and deuterated) were measured using a DMA 5000 Anton Paar vibrating tube densimeter equipped with a temperature controller (peltier device) with a precision of ±0.001 °C and an overall density precision of ±0.00001 g cm^−3^. After careful injection of the sample in the densimeter (assuring that no bubble was left inside the vibrating tube), the temperature scan was set typically from 0 to 7 °C with a temperature step of 0.1 °C in the case of H_2_O and from 7 to 15 °C with a similar temperature step in the case of D_2_O. At least 70 data points in this temperature range were registered for each sample. In the cases where the TMD of the H_2_O solutions were found to be lower than 0 °C, the freezing point depression for the corresponding solution was calculated and the lower value of the temperature range was set accordingly (in order to avoid freezing inside the vibrating tube). The D_2_O solutions did not present such problem due to the larger difference between the TMD and the freezing point temperature of pure D_2_O. Typically each temperature scan took around 4–5 h to complete, with the densimeter placed in a room thermostated at 15 °C in the case of the H_2_O runs and at room temperature for the D_2_O experiments.

**Table 1:** List of ionic liquids used in the present study along with their stated purity and suppliers.

Ionic liquid, origin, purity	Acronym	
1-ethyl-3-methylimidazolium acetate, Iolitec, 95%	[C_2_mim][CH_3_COO]	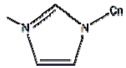
1-ethyl-3-methylimidazolium ethylsulfate, Iolitec, 99%	[C_2_mim][C_2_H_5_SO_4_]; [C_2_mim] ≡	
1-ethyl-3-methylimidazolium tetracyanoborate, Merck, 98%	[C_2_mim][B(CN)_4_]	

## 3. Results

**Figure 1:**
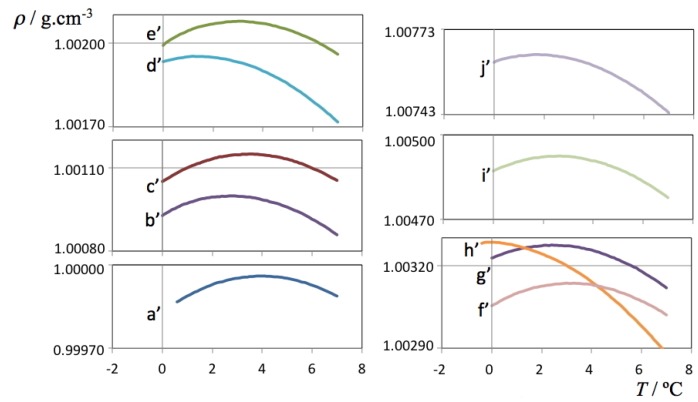
Density as a function of temperature for each of the studied ionic liquid aqueous (H_2_O) solutions. The *a’* label refers to pure water, the *b’-j’* labels refer to the H_2_O solutions listed in Table 2.

**Figure 2:**
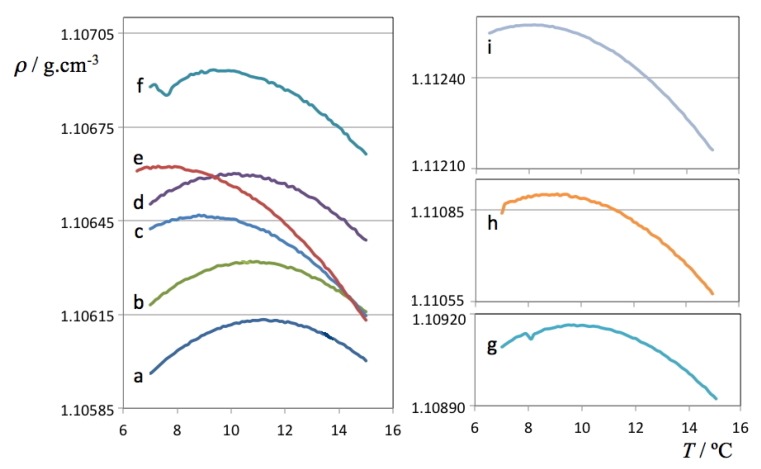
Density as a function of temperature for each of the studied ionic liquid aqueous (D_2_O) solutions. The *a* label refers to pure deuterated water, the *b-i* labels refer to the D_2_O solutions listed in Table 2.

The density data for the H_2_O and D_2_O solutions are presented in Figure 1, Figure 2, respectively. The figures are able to depict on one hand the large amount of data used to determine each TMD (each line comprises 70 or more independent data points whose density was recorded only after equilibration at each set temperature) and on the other hand the required precision of the measuring instrument—although all results are comprised between 0.9998 and 1.0100 g cm^–3^ for H_2_O solutions and between 1.1059 and 1.1126 g cm^–3^ for D_2_O solutions, it was necessary to cut and expand the y-scale into several segments in order to visualize the parabolic curvature of each run around the TMD of each solution.

The obtained density values were fitted to second order polynomials to obtain the corresponding temperatures at the maximum density of each run. Data exhibiting small density fluctuations at the start of some of the runs with D_2_O solutions were not considered. The values obtained for the TMD of pure water and heavy water are 3.98 °C and 11.25 °C, in excellent agreement with the values reported in the literature [1,3] (3.98 and 11.21 °C, respectively). This confirms that the internal consistency and precision of the volumetric data is adequate for this type of determinations and that the calibration of the densimeter thermostat (with an estimated accuracy of only ±0.05 °C) will not impact the Δθ results negatively.

**Table 2:** TMD results for all studied ionic liquid aqueous solutions. Solutions were prepared in molal concentrations. The alphabetical labels correspond to those in Figure 1, Figure 2.

Solute (IL) (*V*_m_/cm^3^mol^−1^)	Solvent (H_2_O)			Solvent (D_2_O)		
	mol/kg	TMD (°C)	Δθ (°C)	mol/kg	TMD (°C)	Δθ (°C)
[C_2_mim][C_2_H_5_SO_4_] (192)	0.061 f’	3.169	−0.816	0.061 g	9.813	−1.437
	0.097 i’	2.590	−1.395	0.098 h	8.882	−2.368
	0.157 j’	1.740	−2.245	0.135 i	8.036	−3.214
[C_2_mim][CH_3_COO] (155)	0.056 c’	3.490	−0.495	0.042 b	10.804	−0.446
	0.104 e’	3.046	−0.939	0.100 d	10.143	−1.107
	0.166 g’	2.439	−1.546	0.162 f	9.667	−1.583
[C_2_mim][B(CN)_4_] (218)	0.032 b’	2.820	−1.165	0.055 c	8.972	−2.278
	0.067 d’	1.431	−2.554	0.097 e	7.408	−3.842
	0.118 h’	-0.413	−4.398			

**Figure 3:**
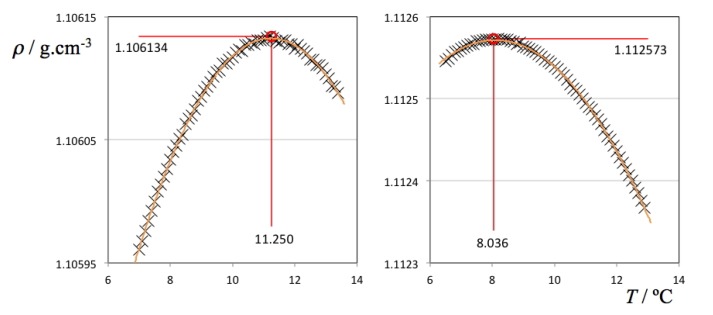
Density as a function of temperature for pure deuterated water (left panel) and a 0.135 molal 1-ethyl-3-methylimidazolium ethyl sulfate aqueous (D_2_O) solution (right panel).

Two selected ρ(*T*) plots from Figure 2 (pure D_2_O and the ionic liquid aqueous (D_2_O) solution with highest density values) are depicted in Figure 3 showing all individual data points, fitting curves and TMD values. It must be stressed that in the case of the H_2_O solutions, the ρ(*T*) parabolic curve could only be measured mostly on one side due to the proximity of the freezing point of the solution on the lower end of the selected temperature range. Nevertheless, in some cases it was possible to measure TMD values for the H_2_O solutions below 0 °C due to the concurrent effect of the freezing point depression of the solutions (cf. left side of the different panels of Figure 1). In the case of the D_2_O there is no such problem: the difference between the TMD and the freezing point temperature of D_2_O is much larger (Δ*T* = 7.4 °C) than that of H_2_O (Δ*T* = 4.0 °C). Nevertheless, and since the temperature runs were done between pre-fixed temperature limits, the parabolic curves obtained in the case of the D_2_O solutions do not have a symmetric distribution of experimental points around the corresponding maxima.

**Figure 4:**
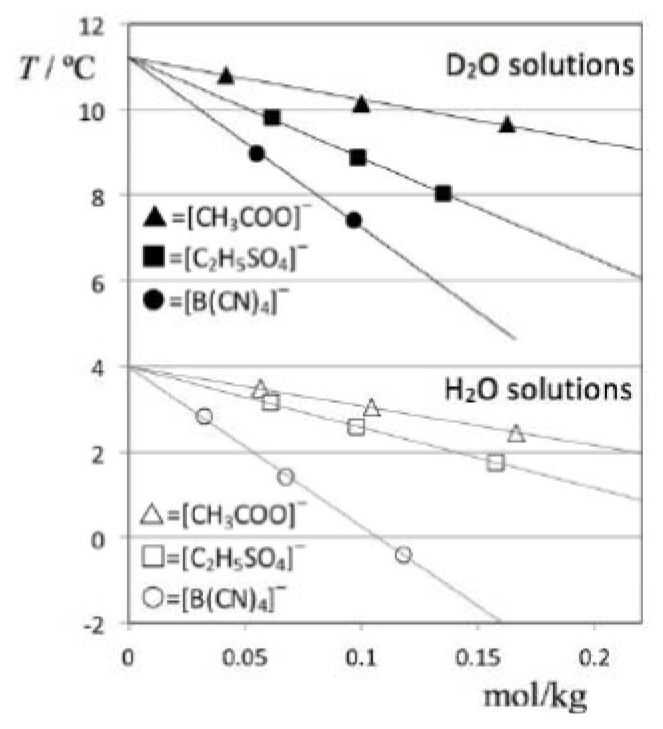
Temperatures of maximum density as a function of molal concentration for D_2_O (filled symbols) and H_2_O solutions (empty symbols) of three different ionic liquids: [C_2_mim][CH_3_COO] = (triangles); [C_2_mim][C_2_H_5_SO_4_] = (squares); and [C_2_mim][B(CN)_4_] = (circles). The anomalous, “isotope-dependent” behavior of [C_2_mim][C_2_H_5_SO_4_] solutions is apparent from the comparison of the D_2_O- and H_2_O-based plots.

The obtained TMD data for all studied solutions is listed in Table 2 and depicted in Figure 4. In order to address the impact of the solute volume in each case (cf. discussion section), the table also includes molar volume values of the pure ionic liquids, estimated at 298K using the predictive method developed by Rebelo *et al*. [7,8].

## 4. Discussion

**Figure 5:**
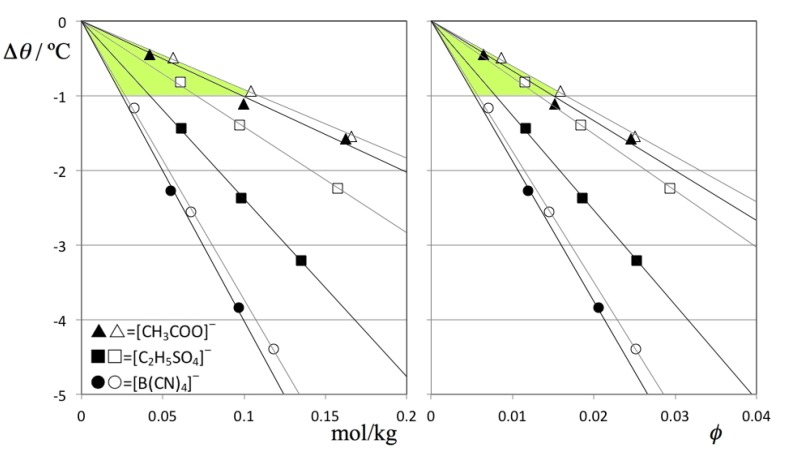
TMD shifts, Δθ, as a function of (A) molal concentration and (B) solute volume fraction, *ϕ*_V_, for D_2_O (filled symbols) and H_2_O solutions (empty symbols) of three different ionic liquids: [C_2_mim][CH_3_COO] = (triangles); [C_2_mim][C_2_H_5_SO_4_] = (squares); and [C_2_mim][B(CN)_4_] = circles. The shaded areas highlight a narrower range of slopes for the B plot, evidencing that a part of the TMD shifts can be attributed to solute volume effects.

Figure 5 shows the TMD shift data (Δθ) for the six solutions under discussion as a function of the corresponding concentrations. The figure shows that the Despretz rule is always obeyed and that the slope of the (Δθ versus molal concentration) lines becomes more negative as the anion of the ionic liquid becomes larger (cf. Table 2).

Previous studies [9] indicate that the size of the solute ions or molecules plays a crucial role in the destabilization of water structure (both H_2_O and D_2_O) in the vicinity of the corresponding TMDs. Such effect is generally much more pronounced than other structure-promoting or structure-disrupting effects that can be observed at higher temperatures: near the TMD almost all water molecules are highly coordinated to each other and part of the tetrahedral patterns found in Ice-I are still partially retained after melting; the introduction of any solute (even if it is able to promote new structures around it) will disrupt the original “open” structure and produce negative TMD shifts. On the other hand, at higher temperatures (where water molecules are less coordinated and the ice-like structure is lost) the same solute will be able to produce net structuring effects.

One way to deduct the effect produced by solutes of different size from the overall TMD shifts is to plot the Δθ values not as a function of molal concentration (Figure 5A) but as a function of the volume fraction occupied by the solute molecules, *ϕ* (Figure 5B). The figure shows that in the present case the size of the anions plays a minor role in the overall trends: the TMD shifts become slightly more similar to each other if the comparisons are performed at equal solute volume fractions instead of equal mole fractions (cf. shaded areas in Figure 5A,B) but the differences in the observed slopes are still noticeable.

This state of affairs is only to be expected since the three ionic liquids selected for this study represent three very different types of behavior towards water: acetate-based ionic liquids are extremely hydrophilic—the acetate anion is the conjugated base of a weak organic acid, which means that in aqueous solution it will be acting as a fairly strong proton acceptor; ethylsulfate-based ionic liquids represent an intermediate case—they are also completely miscible in water in all proportions but the ethyl-sulfate is a weaker base than the acetate ion, which means that it will not be so eager to interact or be included in the hydrogen-bonded network of water; finally, the tetracyanoborate ion is a fairly hydrophobic anion and constitutes the other end of the hydrophilicity/ hydrophobicity range of the trio of anions under discussion (other anions like bistriflamide ([Ntf_2_]^–^) are even more hydrophobic than [B(CN)_4_]^–^ but the corresponding ethyl-methylimidazolium salts are not water-soluble, which precludes any determination of TMD shifts in the corresponding aqueous solutions).

*Hydrophilic versus Hydrophobic anions*. The relation between the hydrophilic/hydrophobic nature of the anion that composes a series of ionic liquids with a common cation and the corresponding TMD shifts has been discussed at length in the work that preceded the present study [4]. Smaller shifts correspond to anions that can interact strongly with the water molecules (hydrophilic) and that at the same time can become part of the extensive hydrogen-bonded network that links the water molecules in the vicinity of their TMD. In this case the negative shifts caused by the disruptive inclusion of the (bulky) ions in the aqueous media are partially compensated by the strengthening of the original hydrogen-bonded network of water by ions that will become (at least partially) part of it. On the other hand very large shifts occur when extremely bulky and more hydrophobic anions are capable of rearranging the water molecules around them without preserving the original open structure of water. Please note that a more complete discussion concerning the long-standing issue of hydrophobic hydration in water [10] is beyond the scope of the present work.

In the case of H_2_O solutions the TMD shifts of three ionic liquids follow the order Δθ([C_2_mim][CH_3_COO]) ≤ Δθ[C_2_mim][C_2_H_5_SO_4_]) << Δθ[C_2_mim][B(CN)_4_]). This means that the strength of both [CH_3_COO]^–^ and [C_2_H_5_SO_4_]^–^ as bases allows them to interact with the hydrogen atoms of water in such a way that they will be able to partially integrate the existing hydrogen-bonded network of the aqueous media. On the other hand [B(CN)_4_]^–^ will interact with water via purely electrostatic (and hydrophobic) interactions that will rearrange the water molecules around the ion without preserving the original hydrogen-bonded network (hence the large Δθ shifts).

When the D_2_O solutions are considered, all Δθ values become larger and the relative order of the effects between the three ions are maintained: Δθ([C_2_mim][CH_3_COO]) < Δθ([C_2_mim][C_2_H_5_SO_4_]) < Δθ([C_2_mim][B(CN)_4_]). However, the relative intensity ratios, namely those corresponding to the [C_2_mim][C_2_H_5_SO_4_] solutions are altered, with the TMD shifts of the [C_2_mim][C_2_H_5_SO_4_] solutions becoming much more similar to those of [C_2_mim][B(CN)_4_]. In other words, whereas the difference between the TMD shifts in heavy and normal water are very similar for the [C_2_mim][B(CN)_4_] or [C_2_mim][CH_3_COO] solutes (slightly more intense in the D_2_O solutions), the difference between the Δθ values in H_2_O and D_2_O solutions is much more pronounced in the case of the [C_2_mim][C_2_H_5_SO_4_] solute (cf. Figure 5B).

Such effect must be, obviously, related to the different nature of the hydrogen bonds in water and heavy water. The question of which is the strongest hydrogen-bonded system is far from trivial and many different authors discussed the problem [11]. The most interesting fact about the whole issue is that the origin of the effect is perfectly understood: the relative energies of H- and D-bonds are caused by differences in their zero-point vibrational energy (ZPVE). However, the problem is that in a condensed phase where different aggregates are present, the energy of the resulting hydrogen-bonded networks must be viewed as an emerging property where cooperative and non-additive effects between multiple species will contribute to different overall outcomes. Nevertheless, *ab initio* studies have shown that the ZPVE of the D bond is lower than that of the H bond in neutral dimers and trimers of water, suggesting more tightly bound structures in the case of heavy water than in the case of normal water [11]. Moreover, another relevant fact for the preset discussion is that pure heavy water exhibits both a higher melting point temperature and TMD than pure normal water. The difference between the melting and maximum density temperatures is also larger in heavy water than in normal water. This means that the open D-bonded network of heavy ice is stronger (more resilient to melting) than the analogous H-bonded network of normal ice.

This state of affairs can help to explain the observed Δθ results between the different H_2_O and D_2_O solutions: in the case of [C_2_mim][CH_3_COO], the strong base character of the acetate anion allows it to continue to act as proton/deuteron acceptor with the water molecules even when they are more tightly bound to each other in the DB network of heavy water. This means that the inclusion of the acetate ion in the original water network will only be slightly less efficient in D_2_O than in H_2_O, resulting in slightly larger TMD shifts in the former solvent solutions. On the other hand, in the case of [C_2_mim][B(CN)_4_] there is no such proton/deuteron acceptor character even for the less tightly bound HB network of normal water (and even less so for the more tightly bound DB network in heavy water). Again, the Δθ results between the different H_2_O and D_2_O solutions of this IL will show only slight differences. Finally, in the case of [C_2_mim][C_2_H_5_SO_4_] solute we have an intermediate situation that will produce a large difference between the Δθ values of the H_2_O and D_2_O solutions: the ethyl sulfate anion is a powerful enough base to get included in the HB network of the H_2_O aqueous solutions but not powerful enough to do the same in the more tightly bound DB network of the D_2_O aqueous solutions. The shifts in the latter case become much more pronounced due to the lack of the stabilizing effect caused by the inclusion of the anions in the DB network of heavy water. In other words the isotope substitution of the solvent exposes the differences in hydrophilicity/hydrophobicity between an extremely hydrophilic anion (acetate), a less hydrophylic anion (ethyl sulfate) and an hydrophobic anion (tetracyanoborate): the first always gets included in the HB/DB structures of both types of water, the second is able to do so in the case of H_2_O and less so in the case of D_2_O, and the third will always be excluded from both HB and DB structures.

## 5. Conclusions

The present work highlights the importance of using isotope substitutions in aqueous media to probe the interactions between water and different solutes.

The analysis of TMD shifts in aqueous solution deals with the same issue. In fact, such type of studies, with solutes ranging from ionic to molecular species, have shown that when a solute is added to water the resulting TMD shifts can be discussed not only in terms of the HB network of water but also how the different possible solute-water interactions—coulomb interactions, hydrogen bonds, hydrophobic effects—must be balanced in order to yield the observed outcome.

Such intricate balance can be shifted by the use of H_2_O- or D_2_O-based solutions. In the present work we were able to reveal in a striking manner the differences in the hydrophilic behavior of three commonly-used ionic liquids, namely the diverse character of [C_2_mim][C_2_H_5_SO_4_] in water and heavy water solutions.
